# Systematic Review of joint preservation limb salvage in osteosarcoma around the knee

**DOI:** 10.3389/fonc.2025.1554799

**Published:** 2025-05-15

**Authors:** Kai Zheng, Xiuchun Yu, Ming Xu, Haocheng Cui, Qian Chen

**Affiliations:** Department of Orthopedics, 960th Hospital of the People's Liberation Army (PLA), Jinan, China

**Keywords:** osteosarcoma, limb salvage, joint preservation, knee, clinical efficacy, complication

## Abstract

**Introduction:**

Joint preservation limb salvage (JPLS) has benefited from advancements in tumor imaging and precision surgical technologies. However, discrepancies exist between the anticipated outcomes of surgical designs and actual clinical results. This study aims to provide a clearer understanding of JPLS.

**Methods:**

A systematic search was conducted across the MEDLINE, Embase, and Cochrane Library databases from January 1, 2003, to December 31, 2023. The search utilized the following keywords: “osteosarcoma,” “bone tumor,” “limb salvage surgery,” “surgery,” “operation,” and “knee.” Inclusion criteria were: (1) publication of original studies in English; (2) clinical research pertaining to JPLS; and (3) studies offering detailed individual patient information.

**Results:**

Ultimately, 25 articles encompassing 224 patients were included. The mean age at diagnosis was 16.8 years (range 2–59 years), with the peak incidence occurring between 9 and 18 years. Male patients predominated, with a male-to-female ratio of 1.46:1. Osteosarcomas were primarily located in the distal femur (170 cases) and proximal tibia (54 cases). Resection lengths were documented for 152 patients, averaging 167.6 mm (range 55–396 mm). Notably, reconstruction methods varied: 76 patients received allograft repair, 90 underwent inactivated tumor bone replantation, and 23 patients had autologous bone reconstruction. Additionally, 35 patients underwent prosthetic reconstruction, with 17 receiving traditionally manufactured customized prostheses and 18 utilizing 3D-printed prostheses. The average Musculoskeletal Tumor Society (MSTS) score for limb function was 26.7 points. Twelve patients experienced local tumor recurrence, 39 succumbed to tumor progression, and there were 96 non-oncological complications, predominantly fractures, infections, and bone nonunion.

**Discussion:**

This review underscores the clinical efficacy of JPLS and examines tumor resection methods, reconstruction techniques, and associated complications.

## Introduction

Osteosarcoma, the most prevalent primary malignant bone tumor in children and adolescents, primarily affects the metaphyses of long bones and poses significant challenges despite advancements in cancer treatment (1, 2). Current 5-year overall survival rates range from 60% to 70% (1). The principal research objectives in osteosarcoma management are to enhance survival rates and improve limb function for long-term survivors. However, survival rates have plateaued, necessitating novel treatment strategies for further enhancement (3). Although sustained improvement in limb function is achievable, it has become a critical focus for surgeons in this field.

Joint preservation limb salvage (JPLS) surgery aims to retain as much healthy joint tissue as possible while completely excising the tumor, and it is gaining recognition within orthopedic oncology (4, 5). Traditionally viewed as high-risk and technically demanding, JPLS has benefitted from advancements in tumor imaging and precision surgical technologies, including computer-aided navigation, 3D-printed surgical guides, and robotic surgery (3, 6). These innovations enhance the safety and precision of JPLS procedures, encouraging orthopedic oncologists to further explore and refine this approach.

In recent years, several reconstructive options for post-oncological JPLS have emerged, with most related research focusing on developments in the past two decades. However, variability among surgical techniques has led to confusion in clinical decision-making. Currently, there is no universally accepted gold standard for reconstruction in JPLS, nor is there a clear clinical algorithm that accounts for individual patient needs in selecting a reconstructive technique. Thus, a systematic literature review on JPLS surgical options is essential to help clinicians understand the advantages and disadvantages of various techniques, facilitating improved clinical decision-making. This review investigates developments in JPLS for osteosarcoma within long bone metaphyses over the last two decades, addressing key questions: (1) Does JPLS increase the risk of tumor recurrence and mortality? (2) What are the optimal methods for bone tumor resection and defect reconstruction in JPLS? (3) What is the functional outcome for patients undergoing JPLS? (4) What are the common postoperative complications of JPLS, and how are they managed?

## Methods

### Information sources and search terms

This systematic review adheres to the Preferred Reporting Items for Systematic Reviews and Meta-Analyses (PRISMA) guidelines. A systematic search was conducted across the MEDLINE, Embase, and Cochrane Library databases from January 1, 2003, to December 31, 2023. The search utilized the following keywords: “osteosarcoma,” “bone tumor,” “limb salvage surgery,” “surgery,” “operation,” and “knee.” Duplicate manuscripts were excluded.

### Study selection and eligibility criteria

Two independent investigators (KZ and HC) conducted a comprehensive review of the title and abstract of all retrieved articles. In cases of disagreement, a full-text evaluation was performed to reach consensus. Studies qualified for inclusion if they met the following criteria: (1) original studies published in English; (2) clinical research on JPLS; and (3) studies providing detailed patient information. Reviews, expert opinions, and conference articles were excluded.

### Data extraction and analysis

Essential attributes from the included studies, such as references, ages, genders, tumor location, reconstruction methods, complications, and knee functional scoring, were tabulated for reference. Patient data extraction was conducted by two independent researchers, with any disputes resolved through discussions among three members of the research team. Concerns were raised regarding potential biases in the studies reviewed, although it was challenging to assess this due to the rarity of osteosarcoma and the uniqueness of surgical methods. The included studies were not randomized controlled trials. While a meta-analysis was considered, the available data did not support its application. A subgroup analysis was conducted to investigate the incidence of complications associated with different reconstruction methods. All analyses were performed using IBM SPSS Version 21 (IBM Corporation, Somers, New York). A p-value of less than 0.05 was considered indicative of a significant association.

## Results

**Figure 1:**
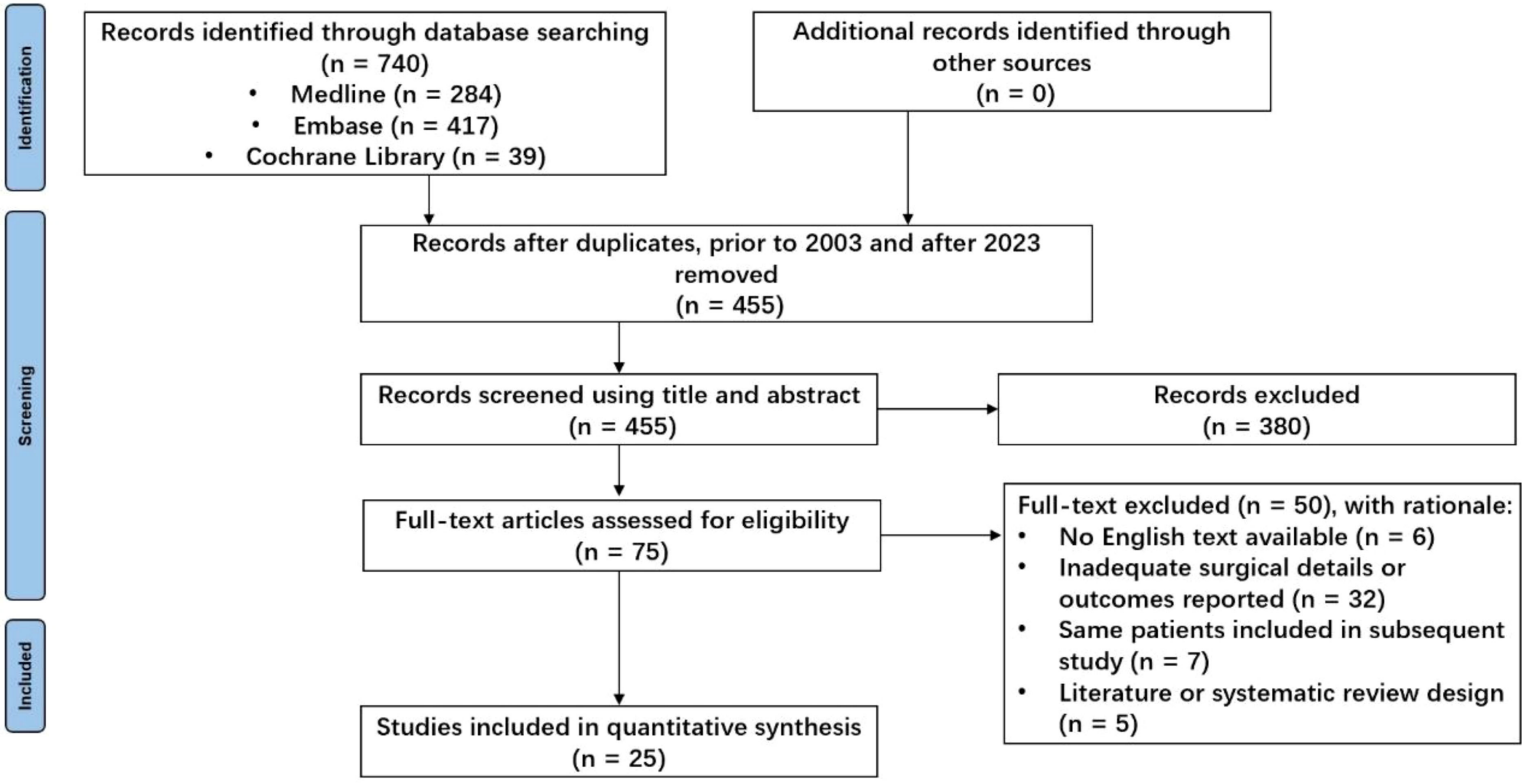
Search results and study selection flowchart.

**Figure 2:**
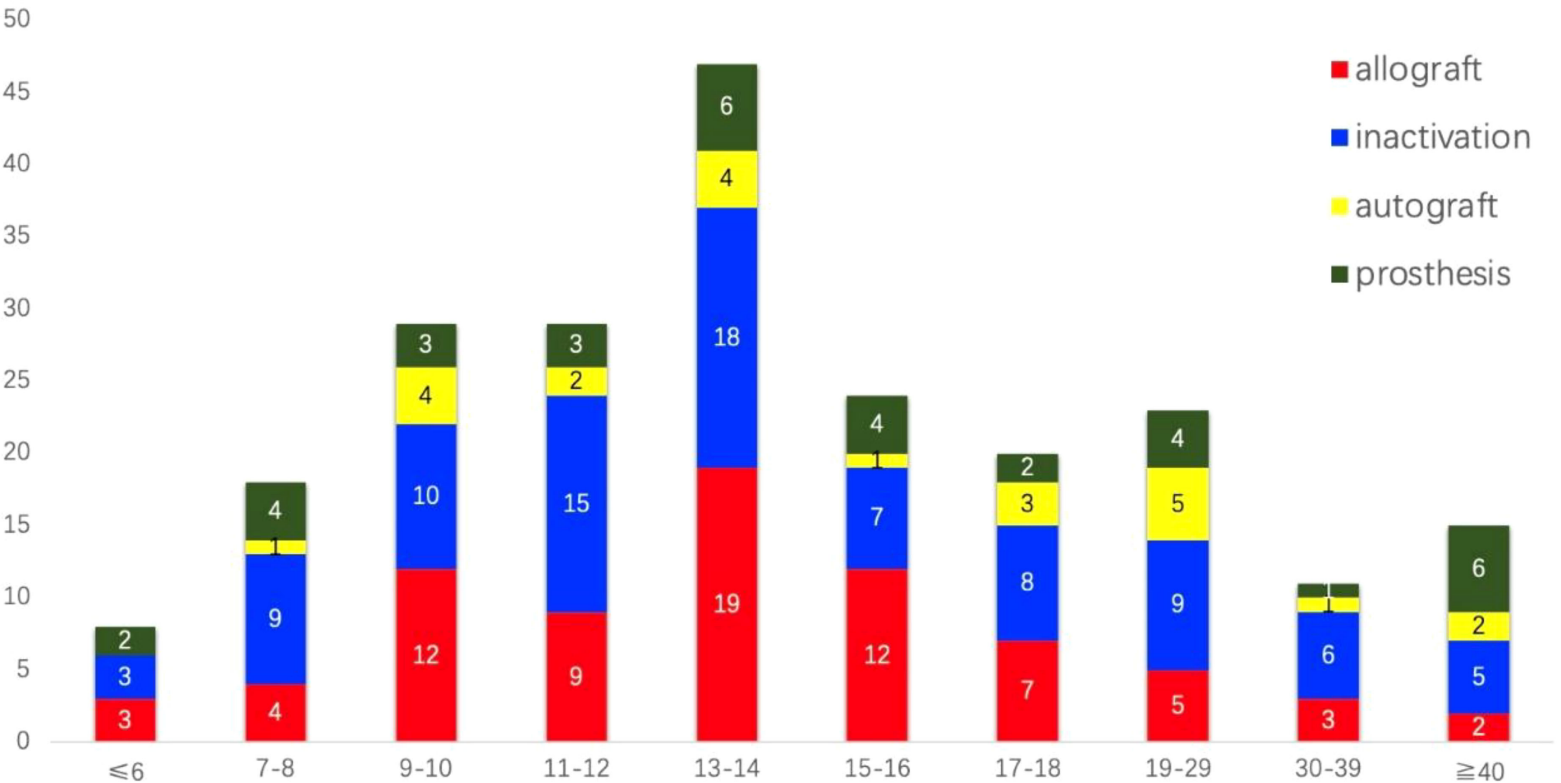
The distribution of age and types of bone defect reconstructions among the 224 patients who underwent joint preservation limb salvage (JPLS) demonstrates significant variability. Patients’ ages ranged from 2 to 59 years, with approximately two-thirds of them belonging to the 9 to 18-year age group.

The initial search identified 740 unique studies, of which 228 were duplicates. After removing duplicates, 380 references were eliminated based on title and abstract screening, and 50 studies were excluded following a full-text review; detailed reasons for exclusion are provided in **Figure 1**. Ultimately, 25 retrospective clinical studies published between 2003 and 2023 were included, comprising a total of 224 patients for systematic analysis (7–31) (see **Table 1**). All studies were observational and retrospective. To be honest, the methodological quality of this study is generally considered average. The age of patients ranged from 2 to 59 years, with a notable concentration between 9 and 18 years (**Figure 2**). The male-to-female ratio was 1.46:1, consistent with typical demographics for osteosarcoma. Of the patients, 170 had tumors located in the distal femur and 54 in the proximal tibia. The average follow-up duration was 78.6 months (range 5 to 313 months).

**Table 1:** The information of 224 patients accepted joint preservation limb salvage (25 studies).

Reference	N	Age Range (Years)	Sex (F/M)	Location (DF/PT)	Reconstruction (N)	Complication (N)	MSTS score Range	Follow-up Range (months)
Gupta et al., 2006 (7)	6	14-21	3/3	6/0	Prosthesis (6)	Infection (1), limited function (2)	20-29	20-31
Jeon et al., 2007 (8)	6	12-52	1/5	3/3	PI (6)	Nonunion (2), infection (1), local recurrence (1)	23-28	38-127
Agarwal et al, 2010 (9)	8	8-13	3/5	6/2	Prosthesis (3), allograft (2), allograft + VF (1), VF (2)	Nonunion (2), local recurrence (1)	27-30	15-66
Yu et al., 2012 (10)	5	6-14	4/1	5/0	AI (5)	Fracture (3)	13-30	60-126
Betz et al., 2012 (11)	4	9-16	2/2	4/0	Allograft (2), VF (2)	Infection (1), nonunion (1)	15-27	18-53
Watanabe et al., 2012 (12)	10	9-46	6/4	6/4	Bone transport (10)	Delayed union (5), fracture (1), infection (1)	25-30	125-237
Puri et al., 2012 (13)	16	8-35	2/14	9/7	II (16)	Infection (3), fracture (2), wound issues (2), vascular injury (1), recurrence (3)	18-30	15-74
Wong et al., 2013 (15)	7	6-46	2/5	5/2	Prosthesis (6), VF (1)	Prosthesis failure (1)	28-30	5-59.8
Kiss et al, 2013 (14)	2	13-14	UN	0/2	VF+NVF (2)	Fracture (1)	–	42-96
Demiralp et al., 2014 (16)	2	19-24	0/2	2/0	Bone transport (2)	Infection (1)	26-27	–
Zhang et al, 2014 (17)	4	9-14	0/4	4/0	Allograft + iliac bone (4)	Nonunion (1)	26 (AVG)	30 (AVG)
Puhaindran et al., 2014 (18)	9	7-13	6/3	7/2	PI + VF (6), PI + NVF (2), VF (1)	Fracture (2), infection (1)	20-30	18-192
Li et al., 2015 (19)	11	9-16	5/6	0/11	Allograft + VF (11)	Infection (1), instability (1), nerve palsy (1), dehiscence (1)	26-30	37-62
Aponte-Tinao et al., 2015 (20)	35	2-50	16/19	26/9	Allograft (35)	Fracture (11), nonunion (3), infection (2), recurrence (3)	10-30	21-276
Lenze et al., 2017 (21)	2	14-18	UN	1/1	NVF (2)	Recurrence (1)	21-28	–
Ikuta et al., 2018 (22)	17	11-58	6/11	16/1	PI+VF (16), PI (1)	Nonunion (7), infection (3), fracture (1), recurrence (2)	14-30	28-198
Campanacci et al., 2018 (23)	9	8-38	2/7	7/2	Allograft + VF (9)	Fracture (3), deformity (1), shortening (1)	25-30	34-313
Takechi et al., 2018 (24)	12	6-14	9/3	8/4	FI (9), FI+VF (1), FI + allograft (2)	Fracture (3), infection (1), recurrence (1)	18-30	41-90
Liu et al., 2019 (25)	15	10-40	6/9	15/0	PI+VF (15)	Fracture (1), shortening (1), osteoarthritis (1)	19-27	31-131
Liu et al., 2020 (26)	11	7-59	4/7	7/4	3D printed prosthesis (11)	Superficial Infection (2)	28 (AVG)	22.5 (AVG)
Kim et al., 2020 (27)	16	6-16	9/7	13/3	Allograft (12), PI (2), FI (1), prosthesis (1)	Nonunion (3), infection (2), fracture (1)	23-30	25-148
Ji et al., 2021 (28)	8	8-16	1/7	7/1	PI + VF (6), PI + NVF (2)	Shortening (2)	25-28	12-52
Wong et al., 2021 (29)	1	16	0/1	1/0	Prosthesis (1)		29	66.8
Smida et al., 2022 (30)	1	13	0/1	1/0	NVF (1)		28	37
Gong et al., 2023 (31)	7	8-15	3/4	7/0	3D printed Prosthesis (7)	Infection (1)	25-30	27-59

N, Number of cases; F/M, female/male; DF/PT, distal femur/proximal tibia; MSTS, Musculoskeletal Tumor Society; PI, Pasteurization inactivation; VF, vascularized fibula; AI, Alcohol inactivation; II, Irradiation inactivation; NVF, non-vascularized fibula; FI, Freezing inactivation; AVG, average.

### Tumor resection

**Figure 3:**
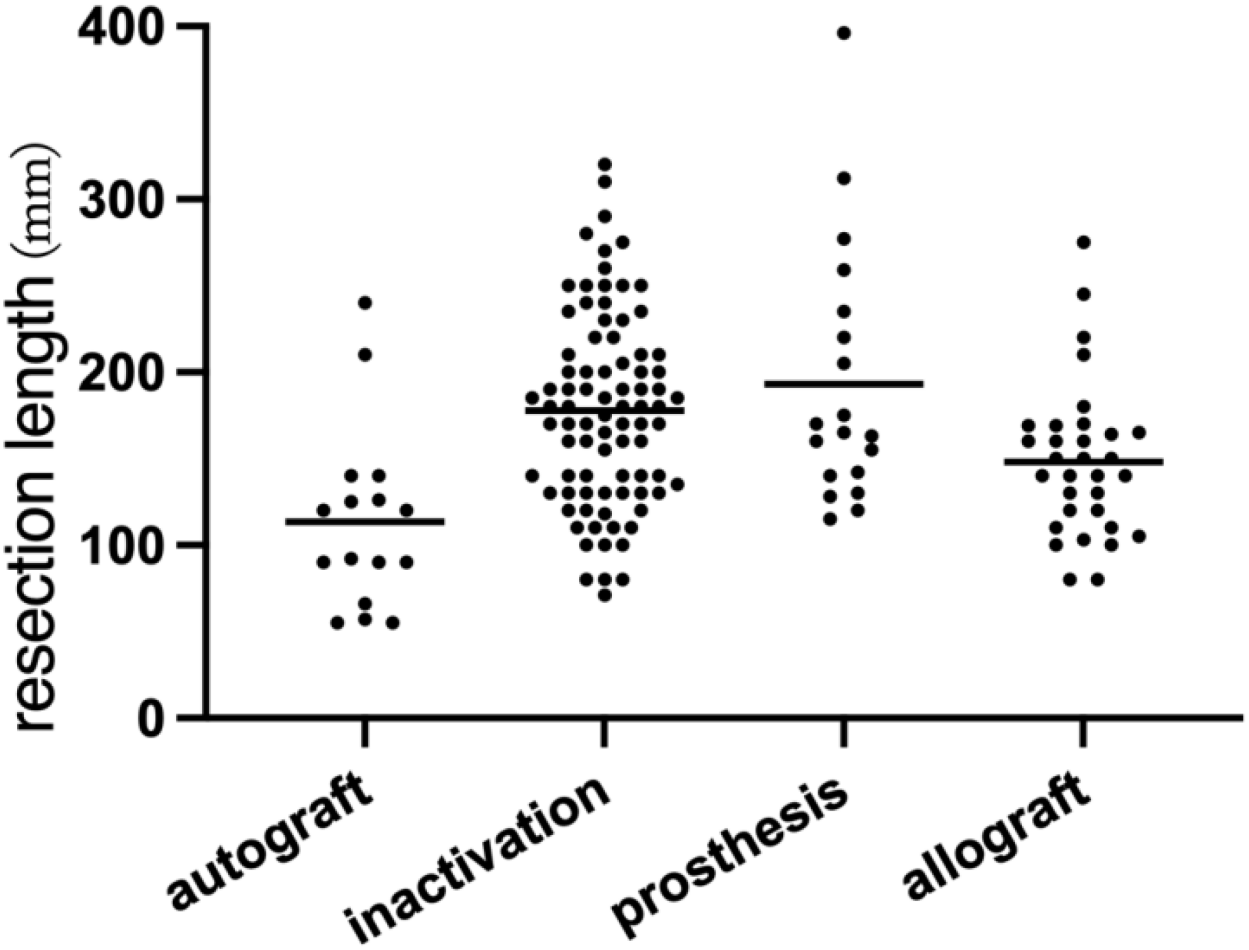
This scatter plot presents data from 152 patients, illustrating the relationship between tumor resection length and the reconstruction method employed. Notably, the bone defect length associated with autogenous bone reconstruction is relatively small. In contrast, the variation in bone defect lengths reconstructed with 3D-printed prostheses is the most pronounced.

Accurate bone resection lengths were documented for 152 patients, averaging 167.6 mm (range: 55–396 mm). The choice of resection length influenced reconstruction methods. Patients with clearly defined resection lengths were visually analyzed through scatter plots according to different surgical techniques (**Figure 3**). Eight patients underwent epiphyseal distraction, and 11 received marginal microwave ablation prior to tumor segment resection; others underwent primary resection. Five patients had partial tumor segment resection using the pedicle-freezing technique, preserving cortical continuity on one side. Navigation techniques, including fused CT-MR image guidance (20 patients) and 3D-printed guide plates (18 patients), facilitated precise tumor segment osteotomy.

### Bone reconstruction

**Figure 4:**
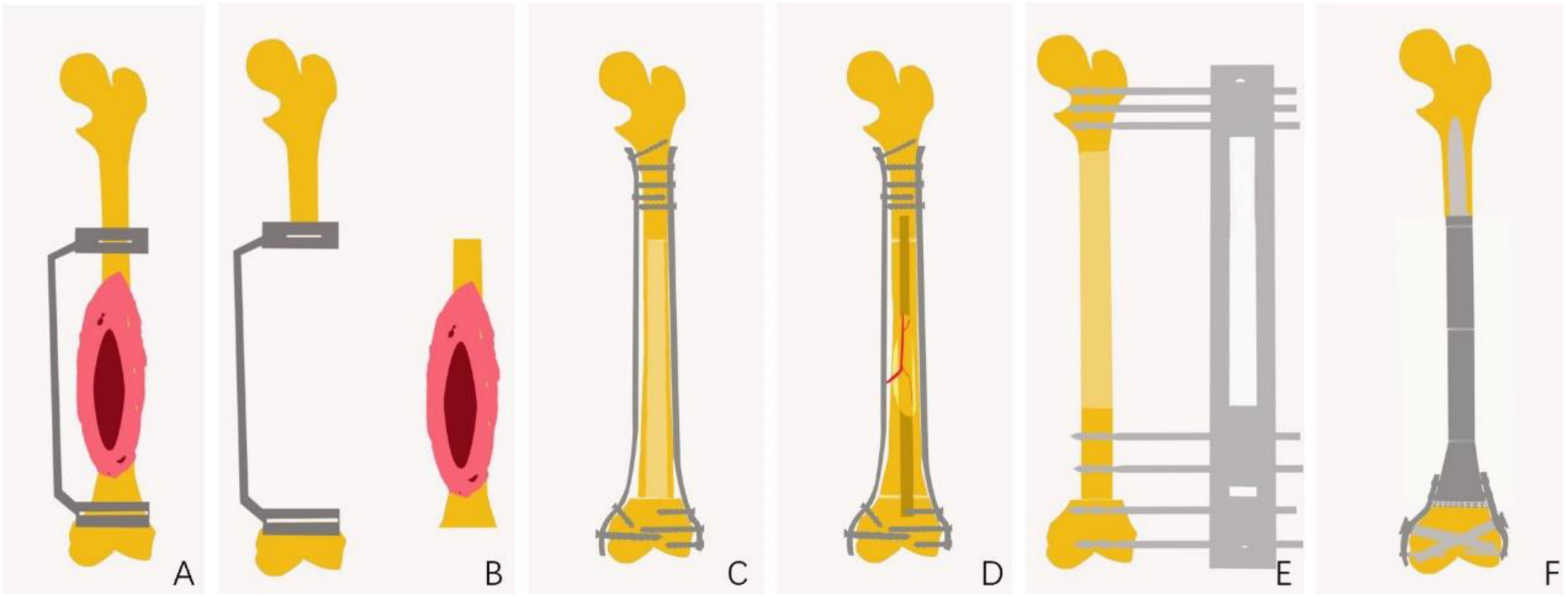
Schematic Diagram of JPLS Surgical Procedure. **(A)** Preoperative design of the osteotomy site, including the design of a 3D-printed customized guide plate to assist in the osteotomy. **(B)** Completion of tumor osteotomy with the assistance of the osteotomy guide plate. **(C)** Allogeneic bone repair for bone defects. **(D)** Inactivation of bone to facilitate repair of bone defects. **(E)** Autologous bone repair for bone defects. **(F)** Customized prostheses for the repair of bone defects.

**Figure 5:**
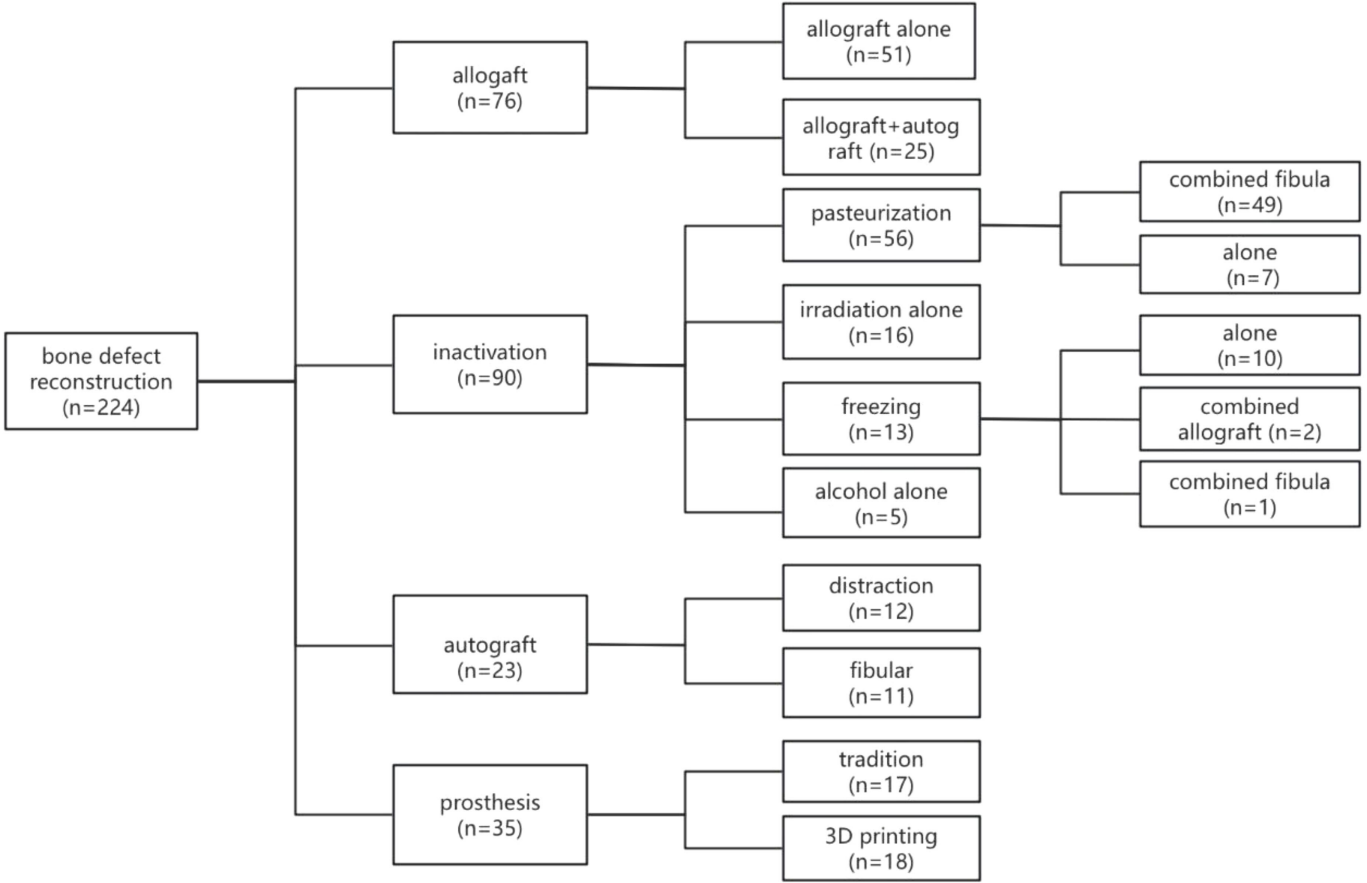
Classification of Bone Defect Repair and Reconstruction Methods Among 224 Patients. The methods for bone defect repair and reconstruction in the 224 patients can be categorized into four main types. Of these patients, 76 underwent allograft procedures, 90 received inactivated tumor bone replantation, 23 were treated with autografts, and 35 were fitted with customized prostheses.

Following tumor resection, methods for bone defect repair and reconstruction among the 224 patients were categorized into four types (**Figures 4**, **5**). Seventy-six patients underwent allograft bone repair, with 25 cases combined with autograft, primarily used alone (51 cases). Ninety patients underwent inactivated tumor bone replantation via various methods: 56 through pasteurization, 16 via irradiation, 13 via freezing, and 5 through alcohol inactivation. Within the pasteurization group, 49 cases combined with fibula, predominantly vascularized (43 cases). The frozen bone group included 2 cases combined with allograft and 1 case with fibula. Neither the irradiation nor alcohol groups combined with any additional methods. Twenty-three patients received autograft reconstruction following tumor resection, including 12 with bone transport, 6 with vascularized fibula, and 5 without. Prosthetic reconstruction was performed in 35 patients, including 17 with traditionally manufactured customized prostheses and 18 with 3D-printed customized prostheses.

### Complications

**Figure 6:**
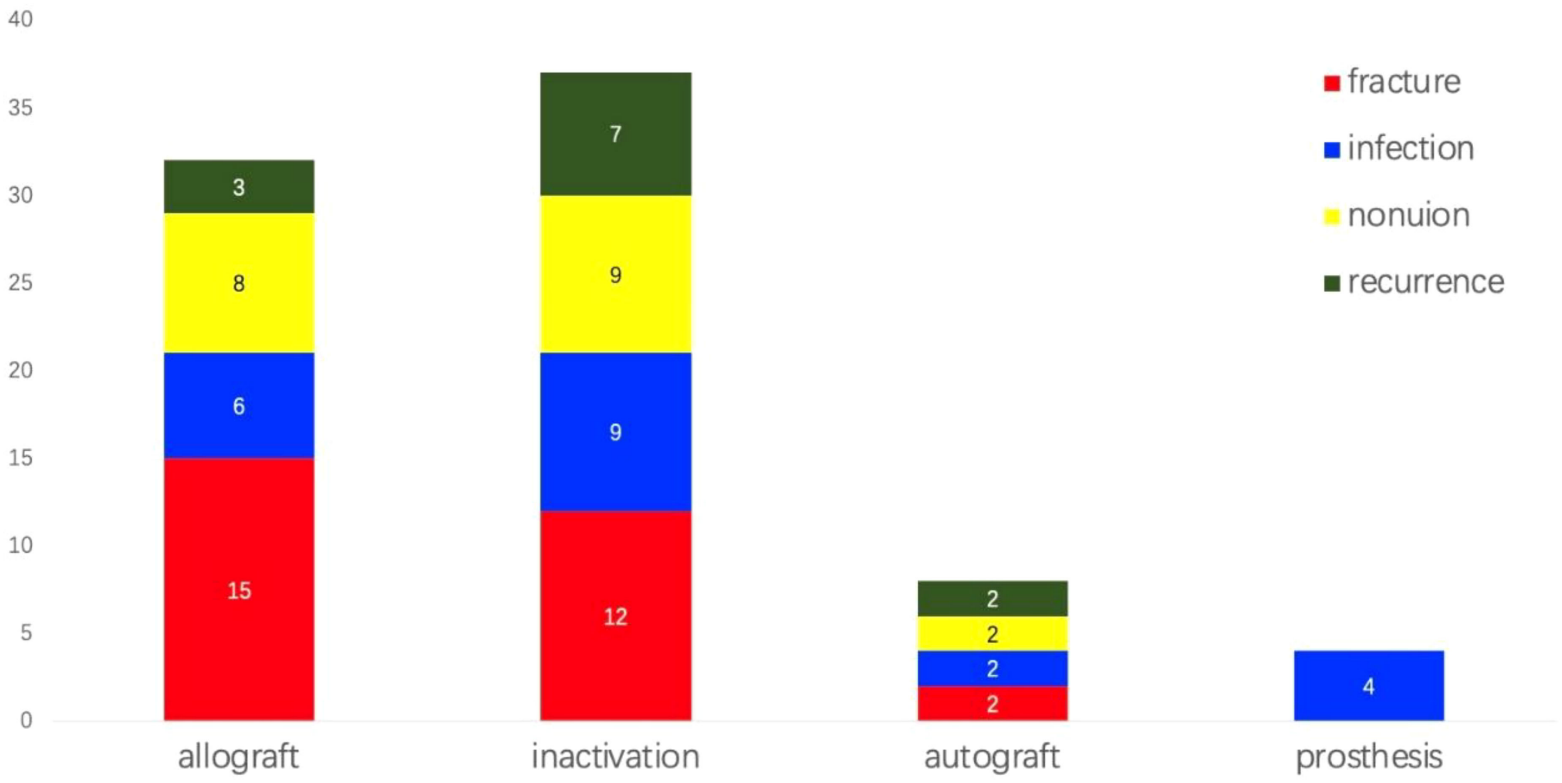
Incidence of complications among patients. Complications were notably observed in the patient population. Twenty-nine individuals experienced bone graft fractures post-surgery, while twenty-one developed infections, and nineteen suffered from bone nonunion. Notably, infection was the only complication observed in patients who underwent prosthetic reconstruction. The overall incidence rates for fractures, infections, and nonunion were 12.9%, 9.4%, and 8.5%, respectively. Additionally, twelve patients experienced soft tissue recurrences.

Complications were significant among patients (**Figure 6**). Twenty-nine experienced bone graft fractures postoperatively, primarily in cases involving allograft reconstructions (15 cases) and inactivation (12 cases). In the allograft group, 12 patients who underwent simple allograft reconstructions and three patients who received allograft combined with autograft experienced fractures. Additional fractures occurred in four patients with pasteurization combined with fibular reconstructions, three with alcohol inactivation, three with freezing inactivation, and two with irradiation inactivation. A bone transport patient and a fibula transplant patient also sustained fractures. The incidence of fractures following autogenous bone reconstruction was 8.7%, in contrast to 19.1% for allogeneic bone and 13.3% for inactivated bone. However, these differences were not statistically significant (p = 0.334). Additionally, a total of 21 cases of infection were reported, including 6 cases associated with allogeneic bone (7.9%), 9 cases with inactivated bone (10.0%), 2 cases with autogenous bone (8.7%), and 4 cases involving prostheses (11.4%). The differences among these groups were also not statistically significant (p = 0.935). Furthermore, 19 cases of bone nonunion were identified: 8 cases with allogeneic bone (10.5%), 9 cases with inactivated bone (10.0%), and 2 cases with autogenous bone (8.7%). Again, no statistically significant difference was observed (p = 0.968). Overall incidence rates for fractures, infections, and nonunion were 12.9%, 9.4%, and 8.5%, respectively. Limb shortening was observed variably, although detailed statistical analysis was not conducted. The average MSTS score for limb function was 26.7 points, with 10 patients scoring below 20, all of whom experienced postoperative complications, some leading to limb loss. Non-oncological complications included abnormal knee joint force lines, knee instability, knee degeneration, bone resorption, and failed internal fixation.

### Oncological outcomes

**Figure 7:**
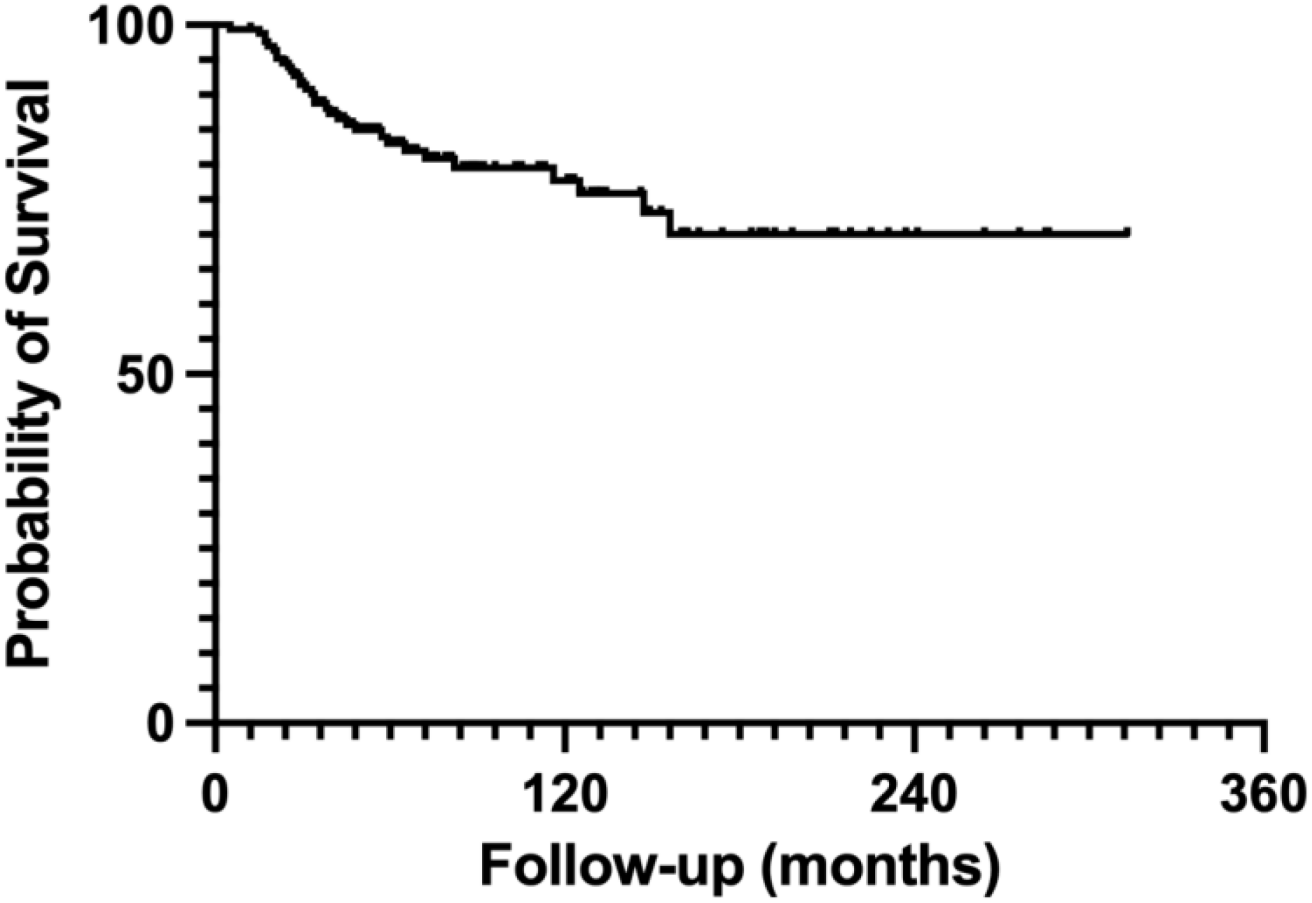
Survival curve for a cohort of 224 patients. The survival curve presented relates to a cohort of 224 patients. Within this group, the 5-year survival rate is 83%, while the 10-year survival rate is 77%.

Oncological outcomes related to tumor recurrence and metastasis revealed that 12 patients experienced local recurrences. Among these, seven patients who underwent inactivation had local recurrences that were not associated with the inactivated autografts; recurrences were identified in the surrounding soft tissues in six cases and within the residual host bone in one case. In the allograft group, no local recurrences occurred in the remaining bony epiphysis, although three patients developed local recurrences in adjacent soft tissues. Additionally, two patients who received autogenous bone transplantation had ambiguous sites of tumor recurrence. These findings suggest that there is no clear relationship between tumor recurrence and the choice of reconstruction method; rather, recurrence is often linked to the resection margin. During the follow-up period, 39 patients died as a result of tumor progression. The 5-year survival rate was 83%, while the 10-year survival rate was 77% (**Figure 7**).

## Discussion

JPLS surgery for osteosarcoma strictly adheres to oncological surgical principles, ensuring complete excision of malignant tissue while maximizing joint function (5, 32). Traditionally, a 2–3 cm tumor-free margin has been deemed necessary; however, advancements in precision surgery, supported by accurate MRI and efficient neoadjuvant chemotherapy, now suggest narrower margins of approximately 1 cm (20, 33, 34). JPLS is built upon these technological developments, which emphasize the preservation of adjacent joints. Surgeons performing JPLS are understandably concerned about tumor recurrence and mortality (35), as survival analyses for osteosarcoma indicate a 5-year overall survival rate of 60-70% (1). This systematic review confirms that JPLS achieves comparable oncological outcomes to traditional limb salvage surgeries, with a tumor recurrence rate of 5.4% and a 10-year survival rate of 77%.

Accurate segmental resection is fundamental to the success of JPLS, and appropriate reconstruction methods are equally critical. Modern technologies, such as computer navigation, 3D printing of guide plates, and robotic surgery, enhance procedural precision and safety (36–40). While the implementation of JPLS requires more energy and financial resources than traditional limb salvage surgery, recent years have seen increasing integration of these technologies into clinical practice, promoting precise tumor resection and defect reconstruction. Among repair and reconstruction methods, inactivated tumor segments are the most common, followed by allogeneic bone, 3D-printed prostheses, and autologous bone. The advantage of inactivated bone reconstruction is that it eliminates the need to consider bone source and compatibility; however, its drawbacks include inappropriateness for patients with severe bone destruction, an increased risk of tumor recurrence, and potential complications such as fractures and non-healing of the inactivated bone. Despite the potential of microwave ablation *in situ*, this study did not include cases involving microwave ablation for osteosarcoma, and concerns remain regarding the absence of standardized procedures for such techniques (41). The advantage of autogenous bone reconstruction is its ability to promote effective bone healing; however, this method is associated with increased trauma and a limited repair length. This variability was validated by scatter plots analyzing resection length and reconstruction types in this study. In contrast, allogeneic bone reconstruction offers the benefit of being unaffected by repair length limitations. Nonetheless, it presents challenges such as allogeneic bone resorption, non-healing, fractures, and the risk of infections. The advent of 3D printing for metal prostheses enhances both surgical precision and bone integration (42, 43). Customized 3D-printed prostheses and guide plates improve the accuracy of resections and defect reconstructions (26, 31). Additionally, the superior bone integration capabilities of 3D-printed prostheses rapidly fulfill patients’ functional needs (44, 45). Nevertheless, ongoing concerns regarding internal stress in 3D-printed prostheses, such as fatigue strength, warrant further investigation (43).

JPLS aims to preserve postoperative limb function, enhancing proprioception and maintaining knee functionality (46). Among the studied patients, those who experienced surgical complications scored below 20 on the MSTS scale, while uncomplicated cases generally achieved normal limb function post-JPLS, restoring levels of physical activity typically limited by other surgical approaches (47). Managing limb length discrepancies post-surgery, particularly critical for pediatric patients, can be addressed with limb lengthening techniques. Some JPLS patients retain limb growth capability, as tumors are situated distant from the epiphyseal plate, preserving its function, whereas others experience compromised growth due to the resection of both the tumor and epiphyseal plate (9).

Surgical complications, including bone graft fractures and nonunion, are prevalent and often interrelated; they are managed with techniques such as POP fixation (28). The pedicle-freezing method shows promise in treating non-healing bones, although its technical complexities hinder broader implementation (24, 48). Notably, 3D-printed prostheses in JPLS exhibit lower complication rates than traditional methods. Infections remain a significant challenge, necessitating focused efforts in the management of large bone defects, whether utilizing inactivated bone, allogeneic bone, or 3D-printed prostheses. Deep infections can be particularly challenging to treat, potentially requiring multiple surgeries or amputations. Effective integration of soft tissue, adequate drainage, and the avoidance of dead space formation are recognized strategies for reducing infection rates. The porous structure of 3D-printed prostheses, promoting improved soft tissue integration compared to traditional machined prostheses, contributes to their increasing acceptance among surgeons, underscoring their role in enhancing JPLS outcomes (49, 50).

The present study acknowledges limitations, including challenges in determining the validity of some included studies, identifying the details of sub-techniques of JPLS, and understanding the impact of certain co-morbidities. While existing literature on JPLS outcomes exists, it is predominantly limited to retrospective, single-arm cohort observational studies, which complicate the assessment of internal validity. Efforts were made to include all relevant articles that could yield useful patient information; however, the sample size remains relatively small. Additionally, inherent risks of publication bias pose challenges to systematic reviews, highlighting the need for caution among readers.

In conclusion, osteosarcoma presents a significant clinical challenge, particularly for younger patients, with profound implications for long-term limb function. Through advancements in pharmacotherapy, precise surgical techniques, and innovations such as 3D-printed personalized prostheses, JPLS represents a promising strategy for enhancing limb function and overall quality of life in osteosarcoma survivors. Continued research and clinical applications of these technologies hold the potential for further improvements in oncological outcomes and patient recovery post-surgery.

## Data Availability

The raw data supporting the conclusions of this article will be made available by the authors, without undue reservation.
